# Autoimmune Enteritis in an Adult With Psoriatic Arthritis, Successfully Treated With Glucocorticoids and Adalimumab

**DOI:** 10.7759/cureus.74965

**Published:** 2024-12-02

**Authors:** Eduardo Avalos, Ali Mohamed, Joel Shapiro, Nida Khalid, Anthony Ocon

**Affiliations:** 1 Internal Medicine, Rochester Regional Health, Rochester, USA; 2 Pathology, Rochester Regional Health, Rochester, USA; 3 Gastroenterology, Rochester Regional Health, Rochester, USA; 4 Rheumatology, Rochester Regional Health, Rochester, USA; 5 Rheumatology, University of Rochester, Rochester, USA

**Keywords:** adalimumab, autoimmune enteritis, chronic diarrhea, etanercept, psoriatic arthritis

## Abstract

Autoimmune enteritis (AIE) is a rare inflammatory condition with intractable diarrhea and malnutrition. Most cases are diagnosed during infancy, but rare adult-onset cases can occur. We present a male patient in his 60s with a history of psoriasis and psoriatic arthritis on etanercept who developed refractory and intractable diarrhea and malnutrition. Serologic and infectious work-up was negative. Despite holding etanercept and attempting treatments with antibiotics, diphenoxylate with atropine, loperamide, octreotide, and pancreatic enzymes, his symptoms persisted, ultimately necessitating total parenteral nutrition. Esophagogastroduodenoscopy showed duodenal bulb ulcerations and colonoscopy was normal. Biopsies showed intraepithelial neutrophils within the duodenal mucosa, active lymphocytic cryptitis, crypt abscesses, and villous atrophy and blunting without granulomas consistent with autoimmune enteritis. The patient was started on prednisone, budesonide, and adalimumab with improvement. After 4 months, he was maintained on adalimumab monotherapy, his bowel habits normalized, and he was able to tolerate a normal diet. This case illustrates a rare presentation of autoimmune enteritis in a patient with well-controlled psoriatic arthritis with etanercept.

## Introduction

Autoimmune enteritis (AIE) is a rare inflammatory condition exemplified by severe, intractable diarrhea. Most cases are diagnosed in infancy in the setting of genetic immunodeficiency and autoimmunity, but spontaneous adult cases do occur infrequently [1-3]. Diagnosis is challenging in adults, given that the incidence is 1/100,000 individuals. Diagnosis criteria include at least six weeks of diarrhea, malabsorption, blunting of small bowel villi, inflammatory lymphocytic deep crypt infiltrate with crypt apoptotic bodies but minimal intraepithelial lymphocytes [1, 2]. Other causes of villous atrophy, such as celiac disease, inflammatory bowel disease, and graft-versus-host disease, need to be excluded. Some patients develop anti-enterocyte or anti-goblet cell antibodies, but this is neither sensitive nor specific to the condition [2]. Treatment is challenging and often includes glucocorticoids and a steroid-sparing agent [1-3]. While many agents have been reported, adalimumab has not been. We report a case of autoimmune enteritis in a patient with a long history of psoriasis and psoriatic arthritis successfully treated with glucocorticoids and adalimumab.

## Case presentation

A 60-year-old Caucasian male presented to the hospital with a new onset of five to six episodes of watery diarrhea per day for the past 2 months. His past medical history was significant for psoriasis and psoriatic arthritis with involvement of peripheral joints, axial joints, enthesitis, and skin. This was treated with etanercept and meloxicam as needed for the past 14 years.

The patient described the stools as non-bloody but dark brown in color. He reported no mucus in the stool, and the diarrhea was not associated with eating food. He had nocturnal episodes of diarrhea as well. The diarrhea was accompanied by a weight loss of approximately 14 lbs. in two weeks, abdominal discomfort, malaise, and fatigue. He eliminated lactose and gluten from his diet without improvement in the diarrhea. He denied fever, chills, nausea, vomiting, new rashes, oral sores, chest pain, shortness of breath, or cough. His diarrhea had never subsided since it started. His psoriasis and psoriatic arthritis were controlled without flares in the skin or joints. He denied any sick contacts or recent travel. On physical exam, his abdomen was not tender or distended, with normal bowel sounds. No mass or blood was found on rectal examination. He had mild, small patches of psoriasis without evidence of inflammatory arthritis. He was empirically started on a 10-day course of ciprofloxacin and metronidazole for bacterial diarrhea, but there was no improvement. Etanercept was held.

As his frequent diarrhea continued for over three weeks, gastroenterology performed esophagogastroduodenoscopy (EGD), which showed severe duodenitis in the bulb with multiple ulcerations that extended into the second portion of the duodenum but no masses, polyps, or arteriovenous malformations. *Helicobacter pylori *was negative. NSAID (non-steroidal anti-inflammatory drugs)-induced enteritis was considered. Meloxicam was discontinued, and he started omeprazole 40 mg twice a day without improvement. Over the next month, diarrhea persisted and became more frequent, with over 10 bowel movements a day. He also developed continuous nausea and vomiting and could not tolerate oral intake. He lost 45 lbs. He was admitted to the hospital and found to be malnourished and cachectic. As shown in Table 1, the patient’s initial laboratory, serologic, and microbiologic workup was non-diagnostic beyond hyponatremia and hypochloremia with increased inflammatory markers. Magnetic resonance imaging (MRI) of the abdomen with contrast showed possible pancreatitis, but lipase was low. He could not tolerate multiple oral challenges due to more frequent diarrhea and started total parenteral nutrition. A combination of diphenoxylate with atropine and loperamide was started with minimal improvement in the frequency of diarrhea. Octreotide and pancreatic enzymes were added but did not improve symptoms.

**Table 1:** Laboratory parameters and diagnostic imaging.

	Test	Results (reference range)
General workup	White blood cells	8300/uL (4000-11000/Ul)
	Hemoglobin	14.9 g/dL (13.0-18.0 g/dL)
	Platelets	314000/uL (150000-400000/uL)
	Sodium	130 mEq/L (135-145 mEq/L)
	Chloride	96 mEq/L (98-108 mEq/L)
	Albumin	2.6 g/dL (3.2-4.8 g/dL)
Inflammatory markers	Erythrocyte sedimentation rate	10 mm/h (0-20 mm/h)
	C-reactive protein	29.4 IU/mL (0-1 IU/mL)
Immunoglobulins	IgA	375 mg/dL (70-400 mg/dL)
	IgG	780 mg/dL (700-1600 mg/dL)
	IgM	62 mg/dL (50-300 mg/dL)
	IgE	179 IU/mL (<158 IU/mL)
Pancreatic Function	Lipase	14 U/L (6-51 U/L)
Hormones	Thyroid stimulating hormone	1.14 IU/mL (0.55-4.78 IU/mL)
	24-hour urine 5-hydroxy indole acetic acid	1.6 mg/24h (<9.8 mg/24 h)
	Vasoactive intestinal peptide	50 pg/mL (<75 pg/mL)
	Gastrin	10 pg/mL (<100 pg/mL)
Autoimmune panel	Anti-parietal cell antibody	Negative
	Anti-islet cell antibody	Negative
	Anti-enterocyte antibody	Negative
	Anti-transglutaminase IgA	1 U/mL (<7 U/mL is negative)
	Anti-transglutaminase IgG	1.2 U/mL (<6 U/mL)
	HLA-B27	Negative
Infectious workup	*Giardia lamblia, cryptosporidia*, ova and parasites	Negative
	Enteric PCR *(Shigella* spp, Shiga toxin genes 1 and 2, *Campylobacter* spp, *Salmonella* spp, *Vibrio* spp, Enterotoxigenic *E. coli*, *Plesiomonas shigelloides, Yersinia *spp)	Negative
	Clostridium difficile	Negative
Imaging	CT chest with and without intravenous contrast	There was no lung mass or adenopathy or pleural effusion.
	CT enterography	There was fluid distention of the right colon and distal colon without wall thickening. There was no small bowel distention, wall thickening, mass lesion, or strictures. No abdominal aortic aneurysm. No pneumoperitoneum or ascites. No diverticulitis. No liver lesions.
	MRI abdomen with and without contrast	Mildly dilated, fluid-filled small bowel loops and wall thickening involving the ascending colon. Diffusely decreased T1 signal throughout the pancreas. No gallbladder, spleen, or kidneys abnormalities.

**Figure 1:**
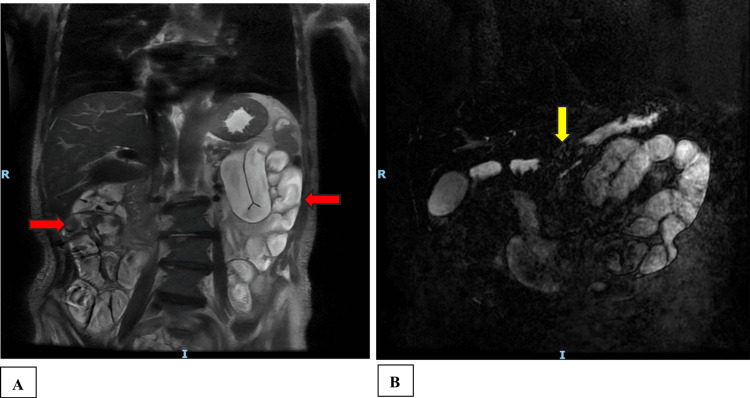
MRI abdomen and pelvis with intravenous contrast In A, there is mildly dilated, fluid-filled small bowel loops throughout the abdomen marked by the red arrows and in B there is evidence of decreased T1 signal throughout the pancreatic body marked by the yellow arrow.

MRI showed mildly dilated, fluid-filled small bowel loops throughout the abdomen and decreased T1 signal throughout the pancreatic body, as shown in Figures 1A, 1B.

**Figure 2:**
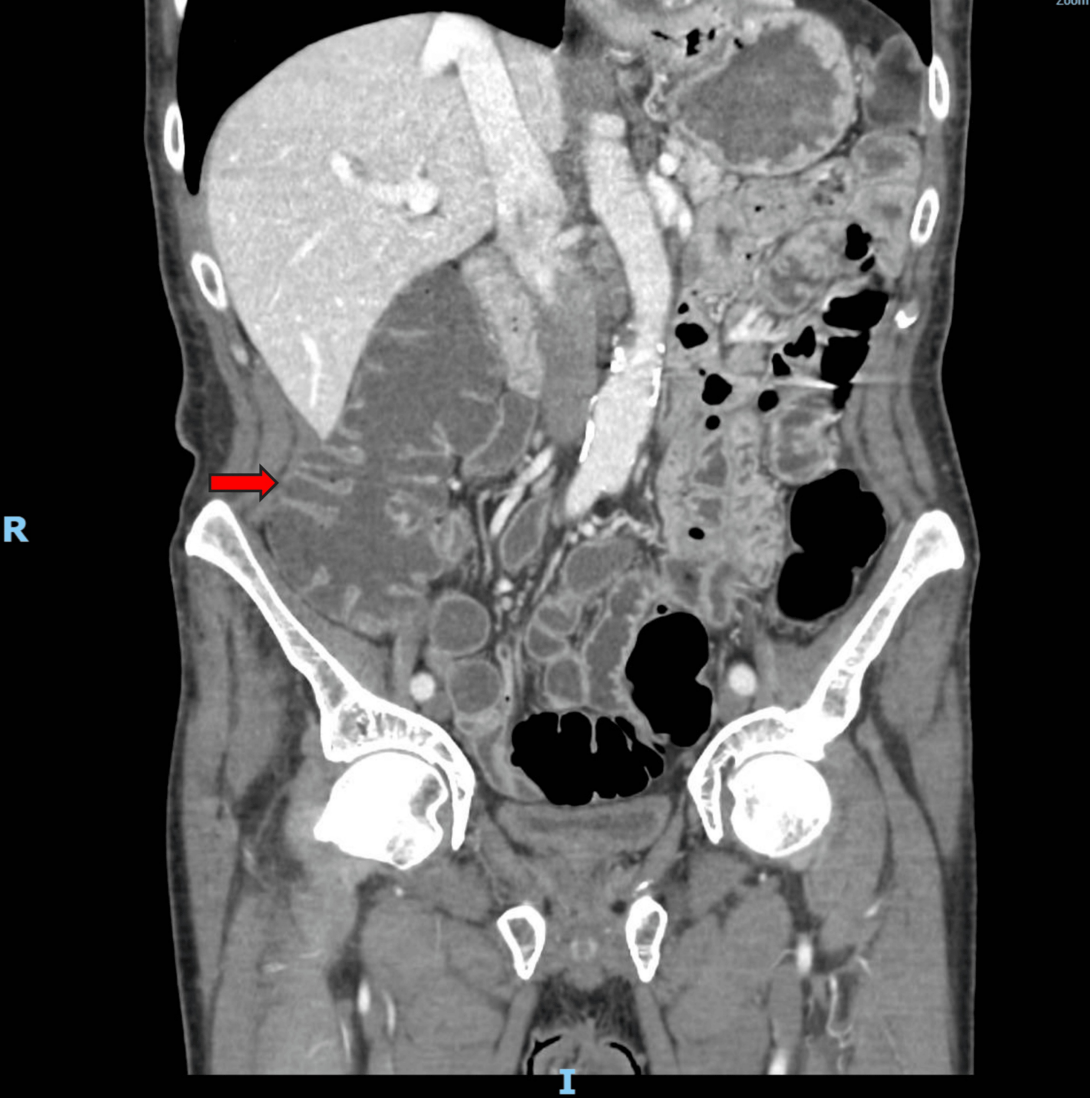
CT of the abdomen and pelvis with intravenous contrast In this CT, there is evidence of fluid in the ascending colon (red arrow).

 Figure 2 shows the evidence of fluid in the ascending colon.

At this time, differential diagnoses included infectious colitis since the patient was on etanercept, inflammatory bowel disease since the esophagogastroduodenoscopy showed ulcers in the duodenum, hormone-secreting tumors, or Whipple disease. Malabsorption syndromes like Celiac disease and Lactose intolerance were less likely since the patient tried avoiding gluten and dairy. Chronic pancreatitis was thought to be less likely since the diarrhea minimally improved with pancreatic enzymes. Microscopic colitis was unlikely since the upper gastrointestinal tract was involved. Irritable bowel syndrome was unlikely since enteral inflammation was found.

**Figure 3:**
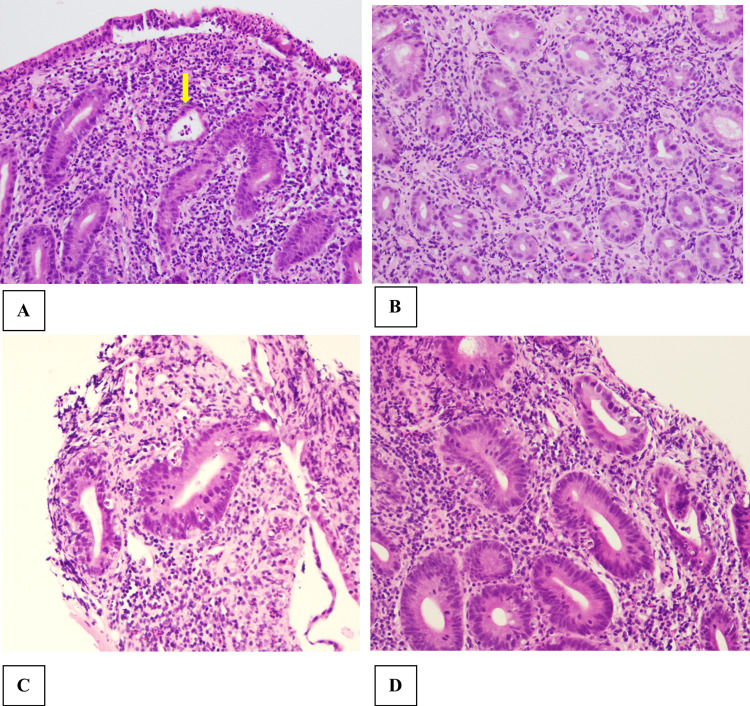
Duodenal and terminal ileum biopsies A) Duodenitis: Duodenal mucosa demonstrating villous blunting with surface intraepithelial neutrophils and lymphocytes. Multiple neutrophils are present within the lamina propria. Globlet cell depletion. A crypt abscess (yellow arrow) (Hematoxilin-eosin staining, 10x) can be seen. B) Duodenitis: Duodenal mucosa demonstrating crypt damage with goblet cell depletion. Intraepithelial neutrophils and lymphocytes can be seen. Apoptic debris can be seen as well (Hematoxilin-eosin staining, 20x). C and D) Ileitis: Damaged terminal ileum showing regenerative type epithelium. Ileal glands with prominent apoptotic debris and intraepithelial lymphocytes (hematoxilin-eosin staining, 20x).

Gastroenterology performed a second EGD, which showed ongoing shallow duodenal bulb ulcers, and a colonoscopy, which was normal. The patient had a video capsule endoscopy, which was largely normal except for erythema, but there were no ulcers, masses, or polyps. Biopsies were taken from the duodenum and the terminal ileum. As shown in Figure 3, biopsies showed intraepithelial neutrophils within the duodenal mucosa, active lymphocytic cryptitis, crypt abscesses, and villous atrophy and blunting without granulomas. PAS (periodic acid-Schiff) stain was negative for microorganisms.

Based on the presentation and symptoms, as well as the villous atrophy on biopsy without other known causes, a previous autoimmune condition, and lack of severe immunodeficiency, the patient was diagnosed with AIE. IBD (inflammatory bowel disease), such as Crohn’s, was less likely due to the video capsule endoscopy not showing any strictures, no granulomas on the biopsy, and lack of large bowel involvement.

The patient was started on intravenous methylprednisolone 20 mg twice daily. Within three days, his bowel movements became less frequent and more formed, and he transitioned to oral prednisone 40 mg daily. Prednisone was slowly tapered, and oral budesonide 3 mg three times daily was added to his regime per Mayo Clinic protocol. Adalimumab 40 mg subcutaneously every 14 days was started as a steroid-sparing agent for autoimmune enteritis but could also treat his psoriasis and psoriatic arthritis. Within a month, the patient had minimal diarrhea, regained his previous weight, and his parenteral nutrition was stopped. After four months, he was no longer on oral steroids or budesonide but maintained on adalimumab monotherapy. His bowel habits normalized. 

## Discussion

We report a case of AIE in a patient who previously had psoriatic arthritis and psoriasis. Our patient was treated with glucocorticoids and then maintained on adalimumab monotherapy. While infliximab has been reported as successful, this is the first report of using adalimumab as a steroid-sparing agent in a patient with AIE with psoriatic arthritis. Adalimumab is United States Food and Drug Administration-approved for treating psoriasis, psoriatic arthritis, and IBD. Given its self-administration and ease of use, our patient preferred it over infusion-based therapy such as infliximab. Our case suggests that adalimumab may be effective for AIE, especially in patients with comorbid conditions, and that it is also approved for treatment.

Prior to AIE onset, our patient was on etanercept for many years to treat his psoriatic conditions. Etanercept does not treat IBD and may actually be associated with a new onset of IBD [4,5]. Etanercept functions as a soluble TNF (tumor necrosis factor) receptor as opposed to a direct TNF-alpha inhibitor such as infliximab or adalimumab. One does wonder whether etanercept may have been implicated in our patient with the onset of AIE. However, this has not previously been reported, and our patient did not improve upon cessation of etanercept. Thus, it is difficult to form a conclusion at this time. The role of TNF-alpha in AIE has not been elucidated and is an area of needed study.

This is the first report of AIE in the setting of prior psoriatic disease. Both psoriatic arthritis and psoriasis are associated with IBD [6]. However, AIE has only been previously reported in one patient with rheumatoid arthritis rather than psoriatic [7]. Other autoimmune conditions are frequently associated with AIE [8]. While the pathophysiology of AIE is not fully understood, alternations in regulatory T-cells via aberrant expression of the FOXP3 (forkhead box P3) gene may play a role [9]. Interestingly, abnormal FOXP3 gene expression and regulatory T-cell dysfunction also may play a role in the pathogenesis of psoriasis and psoriatic arthritis [10,11]. Thus, there is potential for overlapping pathophysiology, and further study of this may yield more insight.

In conclusion, AIE is a rare inflammatory condition that should be suspected in adults who suffer intractable diarrhea and malabsorption. We report a case in the setting of psoriasis and psoriatic arthritis. Our patient responded to glucocorticoids and adalimumab.

## Conclusions

In conclusion, AIE is a rare inflammatory condition that should be suspected in adults who suffer intractable diarrhea and malabsorption. We report a case in the setting of psoriasis and psoriatic arthritis. Our patient responded to glucocorticoids and adalimumab.
